# Absence of CD80 reduces HSV-1 replication in the eye and delays reactivation but not latency levels

**DOI:** 10.1128/jvi.02010-23

**Published:** 2024-02-20

**Authors:** Ujjaldeep Jaggi, Harry H. Matundan, Jay J. Oh, Homayon Ghiasi

**Affiliations:** 1 Center for Neurobiology and Vaccine Development, Ophthalmology Research, Department of Surgery, Cedars-Sinai Burns and Allen Research Institute, Los Angeles, California, USA; University of Toronto, Toronto, Canada

**Keywords:** corneal scarring, CD8, PD-1, virus replication, latency, reactivation

## Abstract

Herpes simplex virus-1 (HSV-1) infections are among the most frequent serious viral eye infections in the U.S. and are a major cause of viral-induced blindness. HSV-1 infection is known to induce T cell activation, proliferation, and differentiation that play crucial roles in the development of virus-induced inflammatory lesions, leading to eye disease and causing chronic corneal damage. CD80 is a co-stimulatory molecule and plays a leading role in T cell differentiation. Previous efforts to limit lesion severity by controlling inflammation at the cellular level led us to ask whether mice knocked out for CD80 would show attenuated virus replication following reactivation. By evaluating the effects of CD80 activity on primary and latent infection, we found that in the absence of CD80, virus replication in the eyes and virus reactivation in latent trigeminal ganglia were both significantly reduced. However, latency in latently infected CD80^−/−^ mice did not differ significantly from that in wild-type (WT) control mice. Reduced virus replication in the eyes of CD80^−/−^ mice correlated with significantly expanded CD11c gene expression as compared to WT mice. Taken together, our results indicate that suppression of CD80 could offer significant beneficial therapeutic effects in the treatment of Herpes Stromal Keratitis (HSK).

**IMPORTANCE:**

Of the many problems associated with recurrent ocular infection, reducing virus reactivation should be a major goal of controlling ocular herpes simplex virus-1 (HSV-1) infection. In this study, we have shown that the absence of CD80 reduces HSV-1 reactivation, which marks the establishment of a previously undescribed mechanism underlying viral immune evasion that could be exploited to better manage HSV infection.

## INTRODUCTION

The majority of viruses are cleared at host mucosal surfaces by the body’s effective innate immune system (1). However, herpes simplex virus (HSV-1) has retained its ability to pose serious threats to infected patients because of its ability to resist clearance from the body after initial infection (2, 3). Current virus eradication treatments include antivirals and/or corticosteroids (4), but these treatments tend to be ineffective once the infection has progressed. HSV-1 has also been referred to as a “sneaky” virus because, after primary infection of epithelial cells, it hides in sensory neurons, thus establishing latency (LAT) (5–7). With the advantage of latency, HSV-1 can be reactivated following any external stimuli, causing tissue damage that creates a crucial need to devise innovative new treatments (8). Previous studies showed that tissue damage is caused by T cells with CD4 T cells as the main orchestrators (7, 9, 10). T cells require two signals to be fully activated and operate properly. The first signal is antigen (Ag) specific and is generated by binding of the T cell receptor (TCR) to Ag-MHC complexes on the Ag-presenting cell (APC). The second, costimulatory signal is generated by the binding of CD28 on T cells to CD80 (B7-1) or CD86 (B7-2) on APC, leading to T cell proliferation, differentiation, and cytokine secretion (11, 12). If the lymphocyte only receives the first signal (i.e*.,* TCR engagement) from the APC, the lymphocyte becomes apoptotic or anergic and is unable to respond to antigen (13, 14).

In previous studies, we showed that HSV-1 suppresses the expression of the costimulatory molecule CD80, but not CD86, in the cornea and that this suppression was associated with the presence of replicating viruses (15). Mechanistically, we found that the HSV-1 ICP22 immediate-early gene binds to the CD80 promoter and that this interaction is required for HSV-1 to suppress CD80 expression (16). We identified ICP22-mediated suppression of CD80 expression in dendritic cells as being central to delaying viral clearance and limiting the cytopathological response to primary eye infection. As a means of survival, we found that HSV-1 uses ICP22 as a mechanism of immune escape that protects the host from increased pathology (16). We also showed that HSV-1-induced CD80 overexpression exacerbated corneal scarring (CS) in BALB/c mice and that a recombinant virus (HSV-CD80) expressed higher levels of CD80 both *in vitro* and *in vivo* (17). CD8^+^ T cell infiltration was enhanced in response to increased CD80 expression by HSV-CD80 virus leading to exacerbated eye disease in infected mice. To further evaluate and understand the impact of ICP22 binding to CD80 on HSV-1 infectivity and pathogenicity, we mapped the region of ICP22 required to bind the CD80 promoter to a 40 aa region of ICP22 and constructed a recombinant HSV-1 with a deletion in this region that does not bind to the CD80 promoter (KOS-ICP22Δ40) (16). The KOS-ICP22Δ40 mutant virus increased CD80 expression in DCs and IFNγ expression in CD8^+^ T cells similar to the ICP22-null virus but did not increase CD80 expression in CD4^+^ T cells in infected mouse corneas.

In the current study, we looked at how the absence of CD80 would affect HSV-1 infectivity by comparing CD80^−/−^ mice with wild-type (WT) control mice. Our results suggest that (i) the absence of CD80 reduced both virus replication in the eye of CD80^−/−^ mice on day 5 post-infection (PI) and gB expression in their trigeminal ganglia (TG) on day 3 PI; (ii) in the absence of CD80, virus reactivation in TG of latently infected mice was slower than in control mice; (iii) latency and survival were similar in CD80^−/−^ mice and WT mice; and (iv) CD80^−/−^ mice had significantly more CD8 T cells and higher PD-L1 expression than WT mice, which did not correlate with higher levels of PD-1 expression in TG of latently infected mice. Overall, our results show that the absence of CD80 does not interfere with disease progression or establishing latency but does help to reduce virus replication in the eye and delays virus reactivation.

## RESULTS

### Absence of CD80 affects virus replication in the eyes of CD80^−/−^-infected mice

**Fig 1:**
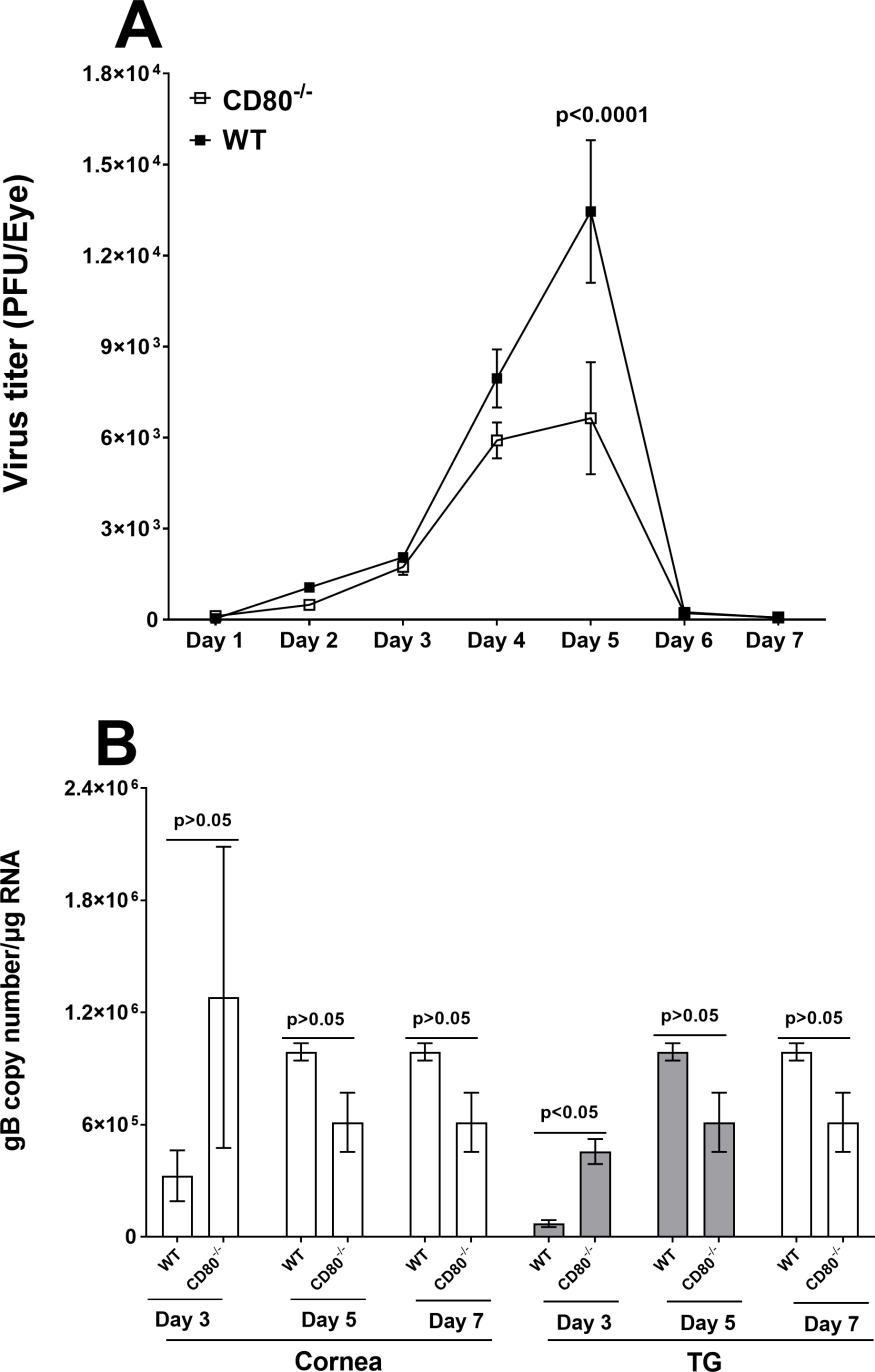
Viral titers and gB copy number in WT and CD80^−/−^ mouse eyes following ocular infection with HSV-1 McKrae strain. (A) Virus replication in infected eyes. WT and CD80^−/−^ mice were infected with 2 × 10^5^ PFU/eye of McKrae virus. The presence of infectious virus in the eyes of mice was monitored daily for 7 days by collecting tear films and quantifying the virus using standard plaque assays as described (see Materials and Methods). Each point represents the mean ± SEM from 50 eyes for both infected mouse groups; and (B) gB copy number during primary infection. Eyes of three WT and three CD80^−/−^ mice were ocularly infected as described above. On days 3, 5, and 7 PI, six corneas and six TG were harvested and the gB copy number was determined by qPCR. No differences in gB copy number were seen between the two groups in either tissue except for significantly higher expression of gB copy number on day 3 PI in TG of CD80^−/−^ mice. The experiment was repeated twice. (A) Virus replication in infected eyes; and (B) gB copy number in infected eyes and TG.

We have previously shown that HSV-1 suppresses CD80 expression in WT mice (15, 16, 18). To directly confirm our previous studies on the effect of CD80 on HSV-1 infectivity, we used CD80^−/−^ mice that lack CD80 expression (19). CD80^−/−^ mice and WT control mice were ocularly infected with 2 × 10^5^ PFU/eye of HSV-1 McKrae. Tear films were collected from 50 eyes per mouse strain on days 1–7 PI and virus titers were determined by standard plaque assays. Virus titers in the eyes of CD80^−/−^ mice were slightly lower than in WT-infected mice until day 4 PI (*P* > 0.05) but were significantly lower in CD80^−/−^ mice than in WT-infected mice on day 5 PI (Fig. 1A, *P* **<** 0.0001). Virus replication eventually dropped down on days 6–7 PI in both infected mouse groups. These results suggest that the absence of CD80 reduces virus replication in the eyes of infected mice, which is a critical factor in HSV-1 pathogenesis.

We next investigated viral glycoprotein gB expression as an indicator of HSV-1 replication using qRT-PCR. CD80^−/−^ and WT mice were infected with 2 × 10^5^ PFU/eye of HSV-1 McKrae as above. Corneas and TG from infected mice were isolated on days 3, 5, and 7 PI and total RNA was isolated as described in Materials and Methods. We found no significant differences in gB expression levels on days 3, 5, and 7 between CD80^−/−^ and WT-infected mice (Fig. 1B, *P* > 0.05). However, the gB copy number was significantly higher on day 3 PI in the TG of CD80^−/−^ mice than in WT mice (Fig. 1B, *P* < 0.05). Although gB copy number was higher in the TG of CD80^−/−^-infected mice than in WT mice and differences on days 5 and 7 in CD80^−/−^-infected mice were lower than in WT mice, but the trend was not significant (Fig. 1B, *P* > 0.05). Overall, in our hands, qRT-PCR data did not replicate the titer results.

### Determining the role of host factors in reducing virus replication in the corneas of infected mice

To determine whether specific host factors correlate with lower virus replication in the corneas of CD80^−/−^ mice, we investigated the role of T cells (CD4 and CD8), innate immune cells (F4/80, CD11c, NK1.1, and LyG6), cytokines (IL-1α and IL-1β, IL-2, IL-4, IL-6, IFNα2, IFNβ, IFNγ, IL-12α, and IL-12β), costimulatory molecules (CD80, CD86, CD28, CTLA4, and CD1d), immune-mediated cytotoxicity molecules (perforin and granzymes A and B), and PD-L1, CD45, and TNF-α. WT and CD80^−/−^ mice were ocularly infected with 2 × 10^5^ PFU/eye of HSV-1 McKrae. On days 3, 5, and 7 PI, corneas and TG were extracted, and total RNA was isolated as described in Materials and Methods. We used a customized panel that included the 28 genes listed above in addition to control genes and performed qRT-PCR as described in Materials and Methods. In both corneas and TG, the expression levels of Ly6G, IL-2, IL-4, IFNα2, IFNβ, IFNγ, IL-12α, IL-12β, CD80, CD86, CD28, CTLA4, and CD1d were not statistically significant on days 3, 5, and 7 (Table S1).

#### T cells

**Fig 2:**
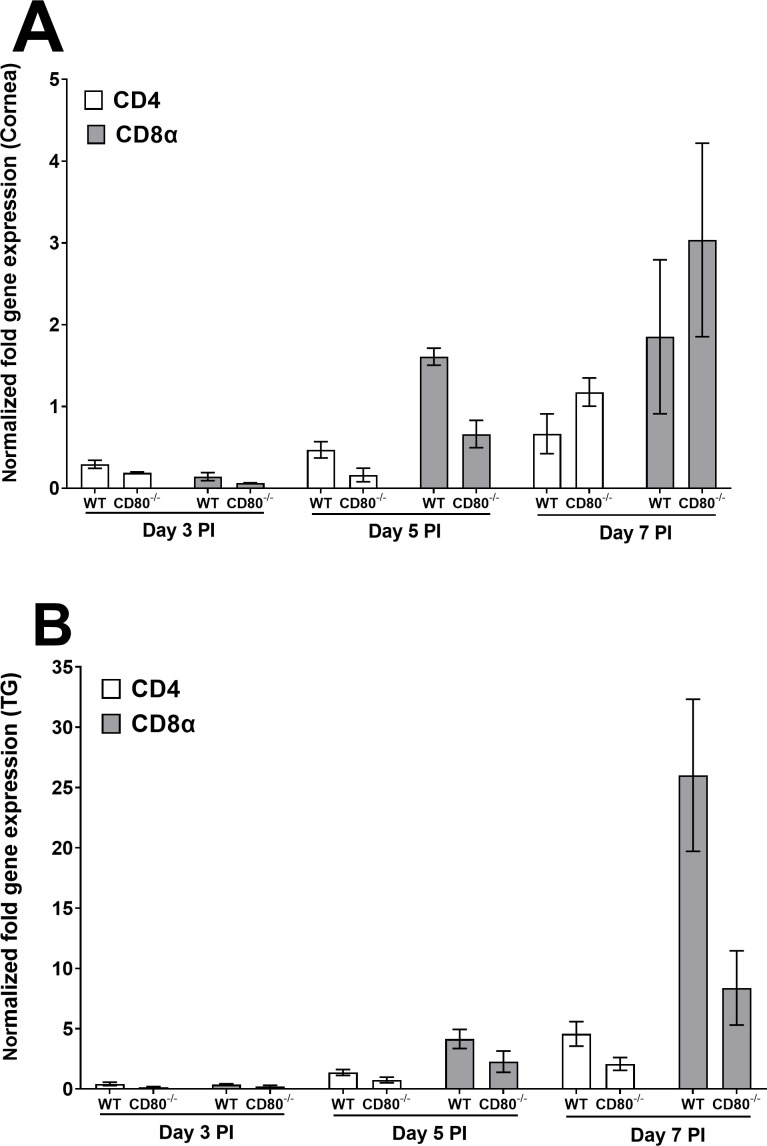
Quantification of CD4 and CD8α RNA transcripts in the corneas and TG of infected WT and CD80^−/−^ mice. (A) Expression in infected corneas. Corneas from WT and CD80^−/−^-infected mice (three mice/group) were harvested on days 3, 5, and 7 PI from mice infected with 2 × 10^5^ PFU/eye HSV-1 McKrae. Total RNA was isolated from each cornea, and GAPDH expression was used to normalize the expression of CD4 and CD8α transcripts in the corneas of ocularly infected mice. No differences in corneal expression were observed between the two infected mice groups. Each bar represents the mean expression ± SEM in six corneas from both infected mouse groups. (B) Expression in infected TG. TG were harvested on days 3, 5, and 7 PI from the above infected mice. Total RNA was isolated from each TG, and GAPDH expression was used to normalize the expression of each transcript in the TG of ocularly infected mice. CD8α expression was lower in CD80^−/−^ mice on day 7 PI (*P* < 0.0001). Each bar represents the mean expression ± SEM in six TG from both infected mouse groups. Only differences that are statistically significant are shown for each gene.

Expressions of CD4 and CD8α were negligible on day 3 PI in both WT and CD80^−/−^-infected mice but increased significantly on days 5 and 7 PI. However, expressions of CD4 and CD8α did not differ significantly between the mouse groups at any time point. CD8α expression was higher in CD80^−/−^-infected mice than in WT-infected mice, but the differences were not significantly different (Fig. 2A, *P* > 0.05). Similarly, we investigated the effect of CD80 absence in the TG of infected WT and CD80^−/−^ mice. On days 3 and 5 PI, expressions of CD4 and CD8α were similar in TG of infected mice (Fig. 2B, *P* > 0.05), but on day 7 PI, CD8α expression in TG of WT-infected mice was significantly higher than in CD80^−/−^ mice (Fig. 2B, *P* < 0.0001). Thus, we can conclude that CD80 expression increases CD8α expression in the TG but not in the corneas of infected mice.

#### Innate immune cell markers

**Fig 3:**
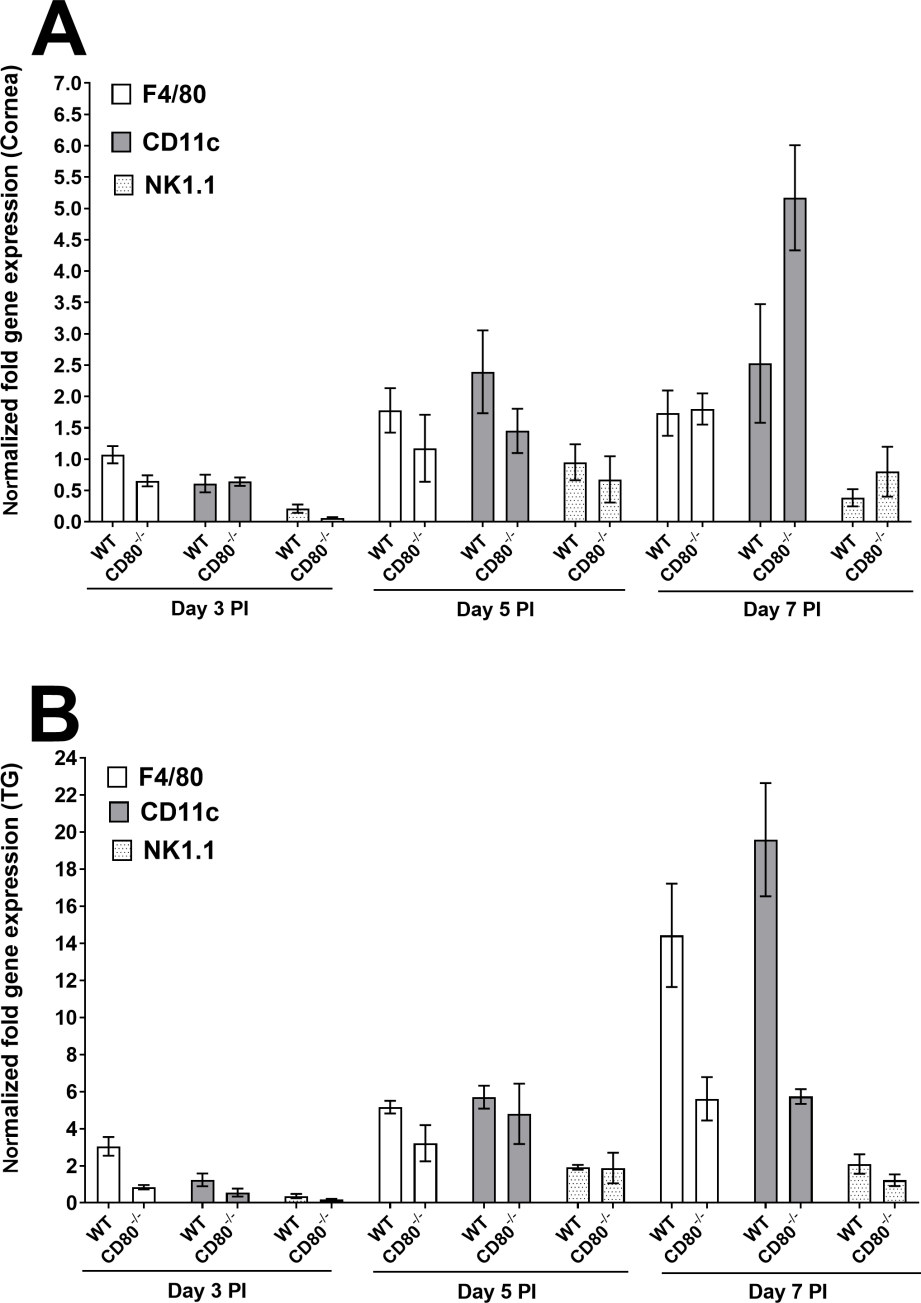
Quantification of F4/80, CD11c, and NK1.1 RNA transcripts in the corneas and TG of infected WT and CD80^−/−^ mice. (A) Expression in infected corneas. WT and CD80^−/−^ mice were infected as described in Fig. 2. Total RNA was isolated from each cornea, and GAPDH expression was used to normalize the expression of F4/80, CD11c, and NK1.1 transcripts in the corneas of ocularly infected mice. CD11c expression was higher in CD80^−/−^ mice on day 7 PI (*P* = 0.0008). Each bar represents the mean expression ± SEM in six corneas for both infected mice groups. (B) Expression in infected TG. TG were harvested on days 3, 5, and 7 PI from infected mice. Total RNA was isolated from each TG, and GAPDH expression was used to normalize the expression of each transcript in the TG of ocularly infected mice. No significant differences in expression were detected among genes in either infected mouse group (*P* > 0.05). Each bar represents the mean expression ± SEM in six TG from both infected mouse groups. Only statistically significant differences are shown for each gene.

We previously showed that HSV-1 downregulates CD80 expression by DCs and not any other cell type (15). To evaluate this finding in our current model, the expression of immune cell markers like F4/80, CD11c, and NK1.1 was examined in the corneas and TG of WT and CD80^−/−^-infected mice. No differences in the expression of genes measured by qRT-PCR were observed between the infected mouse groups, with almost undetectable expression on day 3 PI, and increased expression on days 5 and 7 PI (Fig. 3A, *P* > 0.05). However, on day 7 PI, CD11c expression was significantly higher in CD80^−/−^-infected mice than in WT-infected mice (Fig. 3A, *P* = 0.0008), which confirmed our previous findings (15). We also examined infected TG in WT and CD80^−/−^ mice using the same approach and found no differences in F4/80, CD11c, and NK1.1 gene expressions on days 3, 5, and 7 PI (Fig. 3B, *P* > 0.05).

#### Cytokine expression

**Fig 4:**
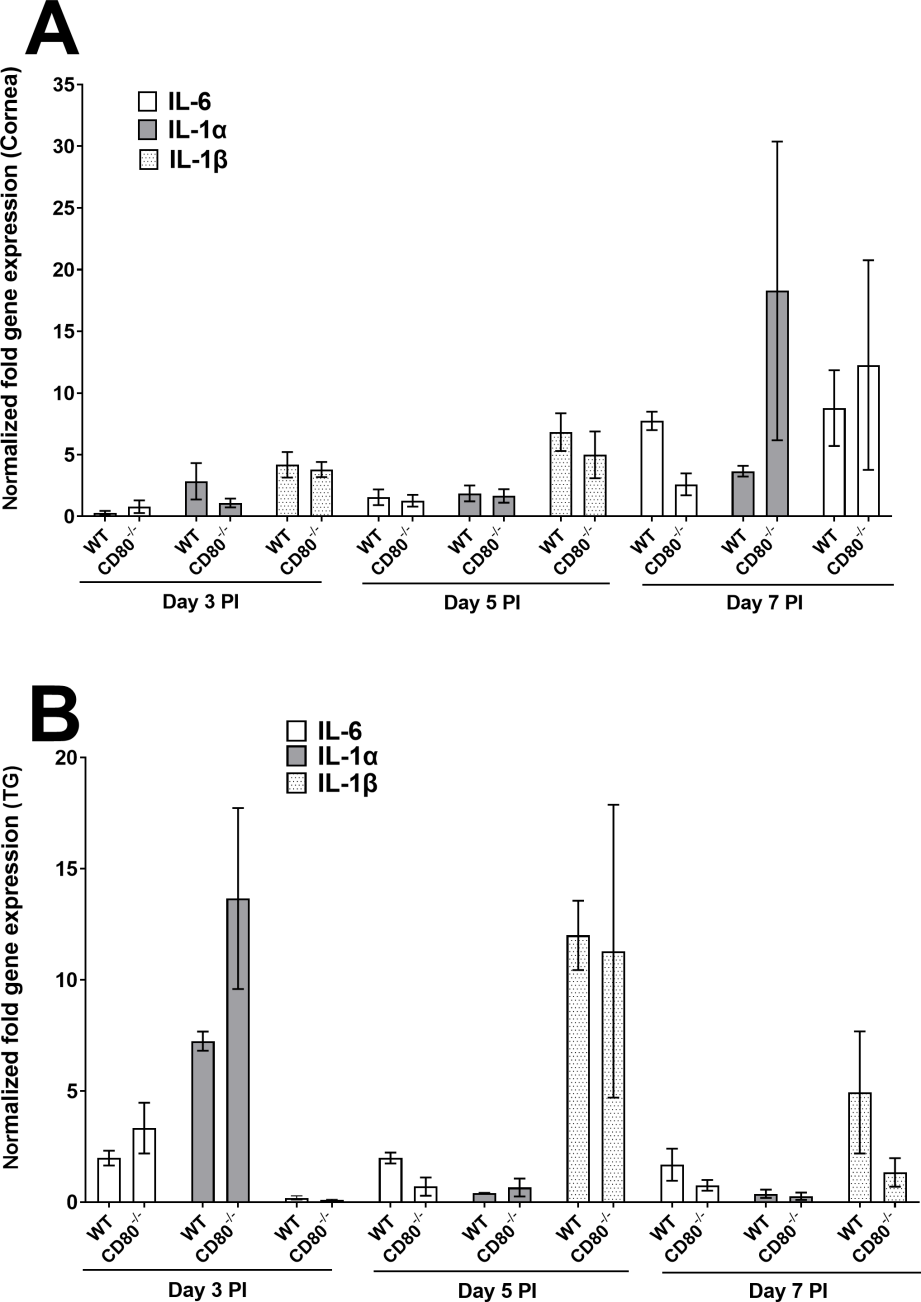
Quantification of IL-6, IL-1α, and IL-1β RNA transcripts in the corneas and TG of infected WT and CD80^−/−^ mice. (A) Expression in infected corneas. WT and CD80^−/−^ mice were infected as described in Fig. 2. Total RNA was isolated from each cornea, and GAPDH expression was used to normalize the expression of IL-6, IL-1α, and IL-1β transcripts in the corneas of ocularly infected mice. No significant expression differences were detected among the genes in the two infected mouse groups. Each bar represents the mean expression ± SEM in six corneas for both infected mouse groups. (B) Expression in infected TG. TG were harvested on days 3, 5, and 7 PI from infected mice. Total RNA was isolated from each TG, and GAPDH expression was used to normalize the expression of each transcript in the TG of ocularly infected mice. Each bar represents the mean expression ± SEM in six TG from both infected mouse groups. Only differences that are statistically significant are shown for each gene.

Since we detected immune cell infiltration into infected corneas and TG, we next investigated possible inflammatory/anti-inflammatory cytokine expressions in the corneas and TG of WT and CD80^−/−^-infected mice on days 3, 5, and 7 PI. Using the same panel as above, IL-6, IL-1α, and IL-1β were nearly undetectable in the corneas of both infected mouse groups on days 3 and 5 PI, but on day 7 PI, some expression was detected with no significant differences between the infected mice groups (Fig. 4A, *P* > 0.05). However, these cytokines were detected in the TG of infected mice but did not differ between the groups (Fig. 4B, *P* > 0.05). Thus, the absence or presence of CD80 does not cause any significant burst in inflammatory cell types after HSV-1 infection.

#### Immune-mediated cytotoxicity molecules

**Fig 5:**
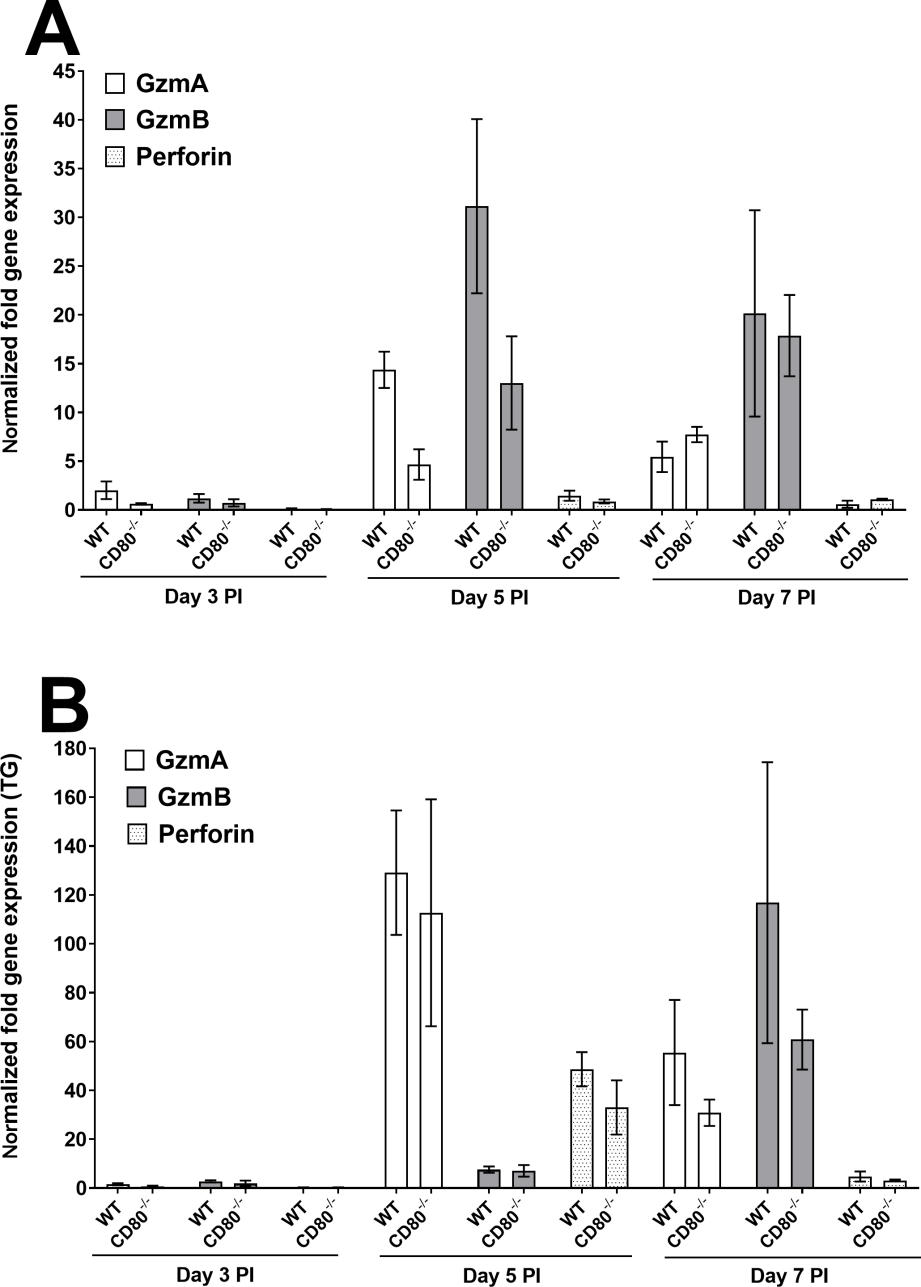
Quantification of GzmA, GzmB, and perforin RNA transcripts in the corneas and TG of infected WT and CD80^−/−-^ mice. (A) Expression in infected corneas. WT and CD80^−/−^ mice were infected as described in Fig. 2. Total RNA was isolated from each cornea, and GAPDH expression was used to normalize the expression of GzmA, GzmB, and perforin transcripts in the corneas of ocularly infected mice. GzmB expression was significantly lower in CD80^−/−^ mice than in WT mice on day 5 PI (*P* = 0.012). Each bar represents the mean expression ± SEM in six corneas from both infected mouse groups. (B) Expression in infected TG. TG were harvested on days 3, 5, and 7 PI from infected mice. Total RNA was isolated from each TG, and GAPDH expression was used to normalize the expression of each transcript in the TG of ocularly infected mice. No significant differences among the genes were observed in infected mouse groups. Each bar represents the mean expression ± SEM in six TG from both infected mouse groups. Only differences that are statistically significant are shown for each gene.

Cytotoxic T lymphocytes and NK cells release granules containing perforin and granzymes at target cells (20). Thus, we investigated the possible effects of granzymes A, B, and perforin expressions on reducing virus replication as described in Fig. 1 using RNA extracted as described above. No significant expressions of GzmA or GzmB were observed in the corneas of infected mice on day 3 PI (Fig. 5A, *P* > 0.05). However, by day 5 PI, expressions of both GzmA and GzmB were significantly higher in the corneas of WT-infected mice than in CD80^−/−^-infected mice (*P* < 0.05), and by day 7 PI, expression levels of both granzymes remained higher but the differences were not statistically significant (*P* < 0.05). In contrast, perforin expression in both groups of infected mice was extremely low on days 3, 5, and 7 PI (Fig. 5A), and granzymes A, B, and perforin expression did not differ significantly in the TG of infected WT and CD80^−/−^ mice (Fig. 5B, *P* > 0.05). Overall, the absence of CD80 did not significantly affect the expressions of granzymes A, B, or perforin in the corneas or TG of infected mice during acute infection.

**Fig 6:**
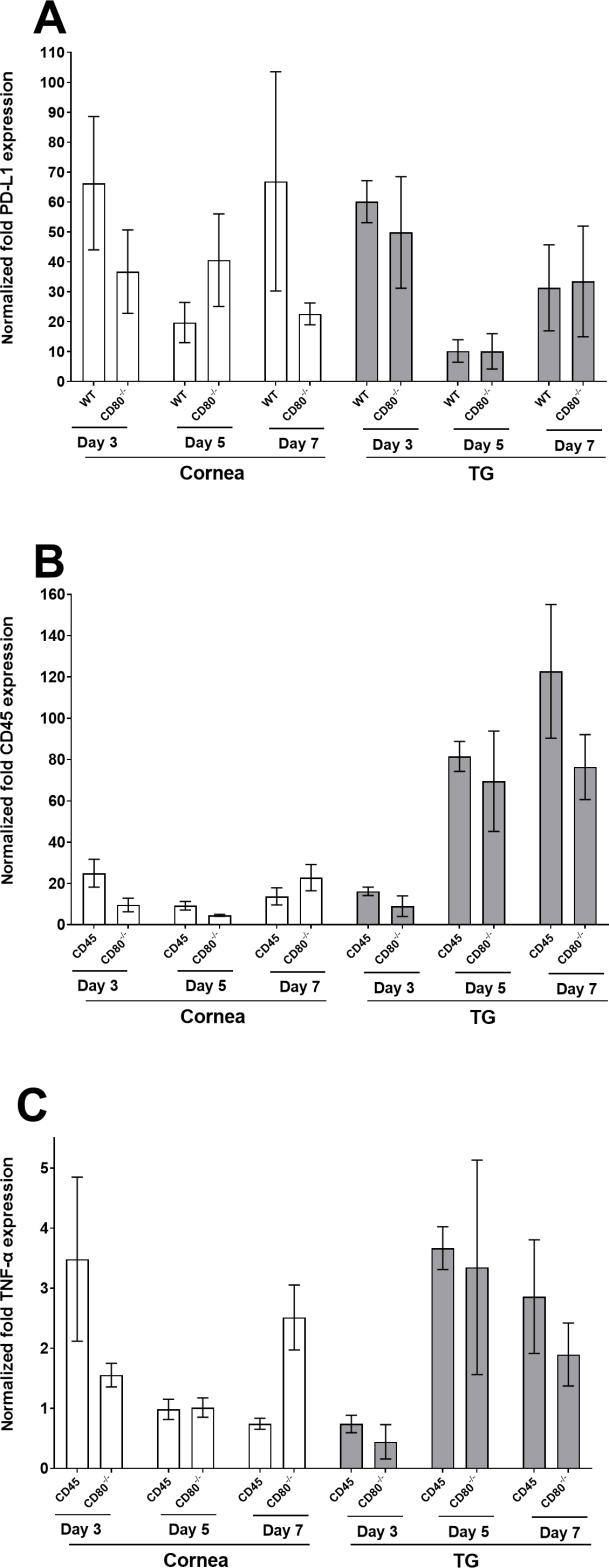
PD-L1, CD45, and TNF-α expression in the corneas and TG of infected WT and CD80^−/−^ mice. (A) Expression of PD-L1 in infected corneas and TG. Corneas and TG from WT and CD80^−/−^-infected mice were harvested on days 3, 5, and 7 PI from mice infected with 2 × 10^5^ PFU/eye of HSV-1 McKrae. Total RNA was isolated from each cornea, and GAPDH expression was used to normalize the expression of PD-L1 RNA transcripts in the corneas and TG of ocularly infected mice. Each bar represents the mean expression ± SEM in six corneas and mean expression ± SEM in six TG. (B) Expression of CD45 in infected corneas and TG. Corneas and TG from WT- and CD80^−/−^-infected mice were harvested on days 3, 5, and 7 PI from mice infected with 2 × 10^5^ PFU/eye HSV-1 McKrae. Total RNA was isolated from each cornea, and GAPDH expression was used to normalize the expression of CD45 RNA transcripts in the corneas and TG of ocularly infected mice. Each bar represents the mean expression ± SEM from six corneas and mean expression ± SEM from six TG. (C) Expression of TNF-α in infected corneas and TG. Corneas and TG from WT and CD80^−/−^-infected mice were harvested on days 3, 5, and 7 PI from mice infected with 2 × 10^5^ PFU/eye HSV-1 McKrae. Total RNA was isolated from each cornea, and GAPDH expression was used to normalize the expression of CD45 RNA transcripts in the corneas and TG of ocularly infected mice. No significant differences were observed among the genes measured in either infected mouse group (*P* > 0.05). Each bar represents the mean expression ± SEM from six corneas and mean expression ± SEM from six TG. Only differences that are statistically significant are shown for each gene.

We also evaluated PD-L1, CD45, and TNF-α gene expression in the corneas and TG of WT- and CD80^−/−^-infected mice. Genes measured by qRT-PCR did not differ between infected mice groups. PD-L1 expression was detected on each day tested but did not differ significantly between infected tissues. (Fig. 6A, *P* > 0.05). CD45 expression was almost undetectable in the corneas on days 3, 5, and 7 PI but in TG, CD45 was slightly upregulated on days 5 and 7 PI but the increase was not significant (Fig. 6B, *P* > 0.05). Similarly, TNF-α expression was detectable in both corneas and TG on days 3, 5, and 7 PI but again did not differ significantly from each other (Fig. 6C, *P* > 0.05). Some of the genes with non-significant levels of expression in both infected mice groups on days 3, 5, and 7 PI are listed in Table S1.

### Absence of CD80 does not affect eye disease or the survival of infected mice

**Fig 7:**
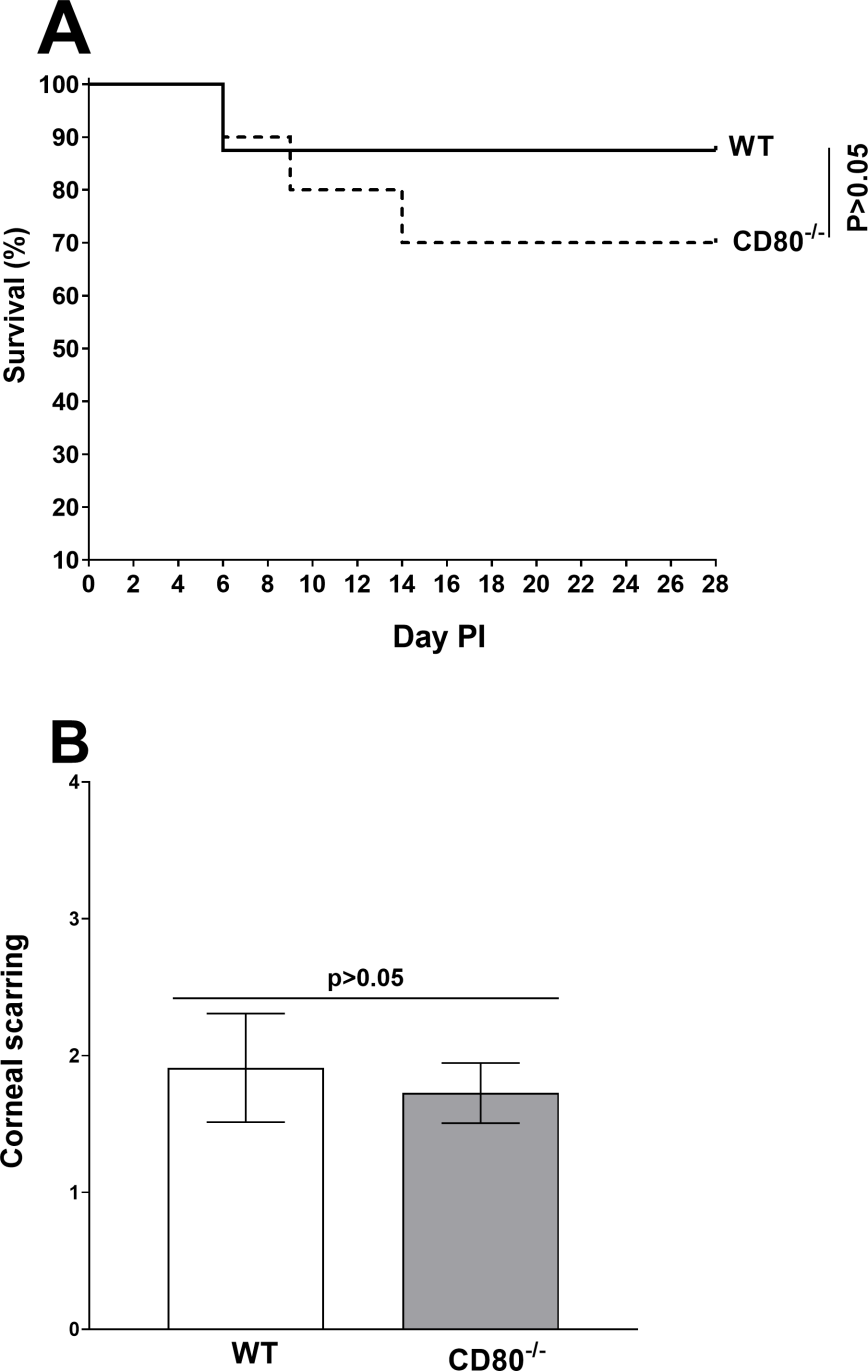
Absence of CD80 does not affect survival and eye disease. (A) Survival. Mice were ocularly infected with 2 × 10^5^ PFU/eye of HSV-1 McKrae as described above. Survival of WT and CD80^−/−^ mice was monitored over a 28-day period after infection. An average of five independent experiments is graphed. (B) Eye disease. A total of 68 eyes from WT and 70 eyes from CD80^−/−^-infected mice used for survival were used to measure CS. Severity of CS in mouse corneas was examined in all groups by slit lamp biomicroscopy. Severity was scored on day 28 PI.

To evaluate the effect of CD80 absence on HSV-1 infection, WT and CD80^−/−^ mice were ocularly infected with 2 × 10^5^ PFU/eye of HSV-1 McKrae as above. We recorded mouse survival and corneal disease for 28 days PI in five separate experiments, and surviving mice were used to measure LAT RNA, gB DNA, and reactivation. Out of 50 mice in the CD80^−/−^-infected group, 35 (70%) survived the ocular infection, whereas 34 of 39 (87%) mice in the WT-infected group survived the ocular infection (Fig. 7A). Although we saw more death in the CD80^−/−^-infected group than in the WT group, these differences were not statistically significant (*P* > 0.05). Levels of CS in surviving mice on day 28 were measured as described in Materials and Methods and were similar in WT and CD80^−/−^ mice (Fig. 7B, *P* > 0.05). These results suggest that CD80 does not play a crucial role in protecting against lethal HSV-1 infection or eye disease in infected mice.

### Absence of CD80 delays reactivation but does not affect latency

**Fig 8:**
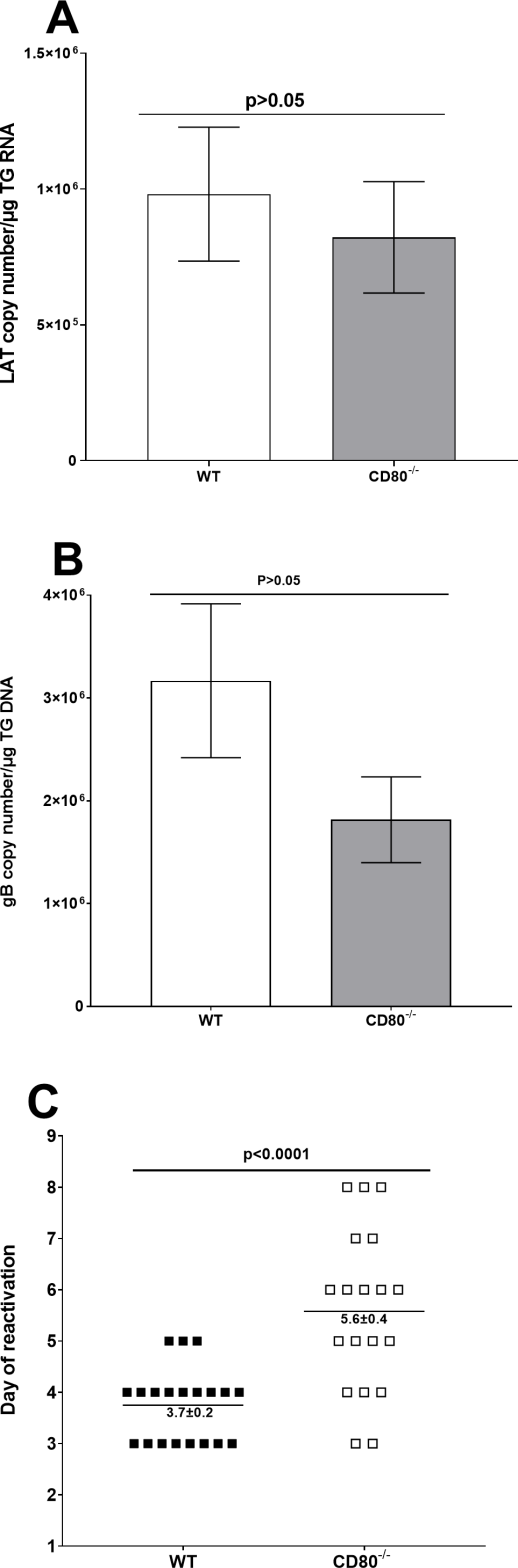
Levels of latency, latent gB expression, and duration of explant reactivation following ocular infection of WT and CD80^−/−^ mice. (A) LAT RNA transcript in latent TG. Eyes from WT and CD80^−/−^ mice were infected with 2 × 10^5^ PFU/eye of McKrae virus. On day 28 PI, TG from infected mice were harvested and LAT expression was analyzed by RT-PCR. qRT-PCR was performed on each individual TG. The estimated relative copy number of HSV-1 LAT was calculated in each experiment using standard curves generated from pGem5317. The plasmid template was serially diluted 10-fold such that 10 µL contained from 10^3^ to 10^11^ copies of LAT. Serial dilutions were then analyzed by TaqMan Real-time PCR with the same probe set. The copy number for each reaction was determined by comparing the normalized threshold cycle of each sample to the standard threshold cycle. GAPDH expression was used to normalize the relative viral LAT RNA expression in the TG. Each bar represents mean copy number ± SEM from 31 TG for infected WT mice and 31 TG for CD80^−/−^ infected mice. (B) gB DNA copy number in latent TG. A total of 11 TG from WT- and CD80^−/−^-infected mice were isolated on day 28 PI. Expression of gB DNA was determined using qPCR, and gB copy number was measured as described in Materials and Methods. (C) Explant reactivation in latent TG. On day 28 PI, TG from infected WT and CD80^−/−^ mice were isolated and incubated in 1.5 mL of tissue culture media at 37°C, and the presence of infectious virus was monitored as described in Materials and Methods. The results are shown as the number of TG that reactivated daily. Each point represents mean reactivated TG ± SEM of 20 TG for WT mice and 19 TG for CD80^−/−^ from two independent experiments. (A) LAT RNA; (B) gB DNA; and (C) explant reactivation.

To determine whether CD80 plays a role in establishing latency, WT and CD80^−/−^ mice were ocularly infected with 2 × 10^5^ PFU/eye of HSV-1 McKrae. On day 28 PI, TG were extracted and the amount of LAT transcript in latently infected individual TG was measured by qRT-PCR in two separate experiments. LAT transcript levels did not differ significantly between the groups (Fig. 8A, *P* > 0.05). We also found that the HSV-1 gB copy number measured on day 28 PI in the TG of latently infected mice was not statistically different between latently infected WT and CD80^−/−^ mice (Fig. 8B, *P* > 0.05). These results suggest that the absence of CD80 expression does not affect the levels of LAT RNA or gB DNA in the TG of latently infected mice.

To further analyze the effect of CD80 absence on reactivation, TG from mice that survived ocular infection were isolated on day 28 PI and monitored for the presence of infectious virus by explant reactivation as described in Materials and Methods. The average time to reactivation in infected WT mice was 3.7 ± 0.2 days, while in infected CD80^−/−^ mice, the time to reactivation was significantly delayed at 5.6 ± 0.4 days (Fig. 8C, *P* < 0.0001). These results suggest that the absence of CD80 expression delays reactivation but does not affect latency.

### Effect of CD80 absence on exhaustion markers and latently expressed genes in latently infected mice

**Fig 9:**
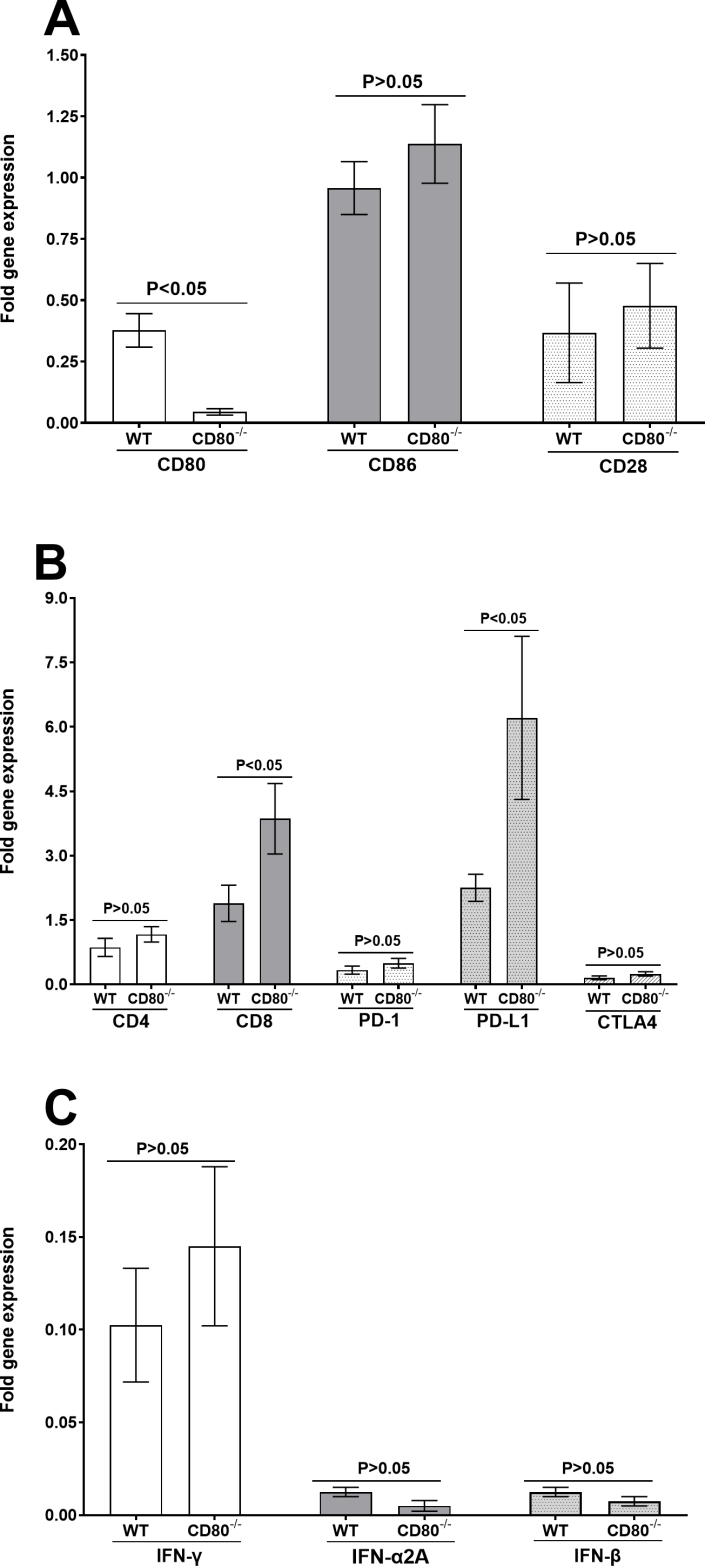
Levels of CD80, CD86, CD28, CD4, CD8, PD-1, PD-L1, CTLA4, IFN-γ, IFN-α2A, and IFN-β. (**A**) Expression of CD80, CD86, and CD28. Mice were infected with 2 × 10^5^ PFU/eye of HSV-1 McKrae as described above. TG from infected mice were extracted on day 28 PI. Total RNA was isolated from each TG, and GAPDH expression was used to normalize the expression of each transcript in the TG of ocularly infected mice. Each bar represents the mean expression ± SEM from 10 TG for each mouse strain. (**B**) Expression of CD4, CD8, PD-1, PD-L1, and CTLA4. RNA extracted from the TG of latently infected mice described above was used to measure the expression of CD4, CD8, PD-1, PD-L1, and CTLA4. Each bar represents the mean expression ± SEM from 10 TG. (**C**) Expression of IFN-γ, IFN-α2A, and IFN-β. RNA extracted from the TG of latently infected mice as described above was used to measure IFN-γ, IFN-α2A, and IFN-β TG expression on day 28 PI. Each bar represents the mean expression ± SEM from 10 TG per mouse strain.

We next asked whether the absence of CD80 has a functional impact on T cell exhaustion. Exhaustion is a fundamental event in many viral infections, and PD-1, PD-L1, and CTLA4 transcript levels are markers of T cell exhaustion (21, 22). RNA isolated from the TG of latently infected mice described above was used to measure the expression levels of CD80, CD86, CD28, CD4, CD8, PD-1, PD-L1, CTLA4, IFN-γ, IFN-α2A, and IFN-β transcripts by qRT-PCR using customized latency assay plates. The results are presented as a “fold” change in transcript level for each gene normalized to GAPDH housekeeping gene expression. Expressions of CD86 and CD28 were similar in the TG of WT- and CD80^−/−^-infected mice (Fig. 9A, *P* > 0.05) despite negligible CD80 expression specifically in CD80^−/−^ mice (Fig. 9A, *P* < 0.05).

We evaluated the expression of CD4, CD8, PD-1, PD-L1, and CTLA4 transcripts in WT and CD80^−/−^ latently infected mice and found that the expression of CD4, PD-1, and CTLA4 did not differ statistically between the two mice groups (Fig. 9B, *P* < 0.05); however, the expressions of CD8 and PD-L1 were higher in CD80^−/−^ mice than in WT-infected mice (Fig. 9B, *P* < 0.05). This result is consistent with our previous study showing that CD80 binds to PD-L1, leading to increased T cell activation (23). We hypothesize that in the absence of CD80, PD-L1 expression is increased leading to a higher number of CD8 T cells with no significant effect on CD4 T cells. We further measured the levels of IFN-γ, IFN-α2A, and IFN-β but found no significant differences in their expression between the two groups of infected mice (Fig. 9C, *P* > 0.05).

## DISCUSSION

Stromal keratitis caused by HSV-1 is an important clinical problem in humans that is managed mainly using anti-inflammatory drugs. It becomes challenging when there are periodic outbreaks, often with lesions forming in infected individuals as a result of virus reactivation. If not controlled, stromal keratitis can cause severe tissue damage and in rare cases, blindness in humans. Tissue damage is mainly caused by T cells (24) but can also involve non-lymphoid cells, particularly neutrophils and macrophages as previously reported (25–27). The extent and duration of ocular disease following HSV-1 infection correlate with the time required for immune clearance of the virus from the eye as well as increased ocular viral loads. Virion production in the eye during acute infection is clinically significant as increased viral load correlates with protracted ocular disease. After primary infection and replication in the eye, HSV-1 establishes latency in the ganglia of infected individuals (28, 29). Mechanisms that maintain latency are of great clinical significance because of serious CS results following HSV-1 reactivation. Indeed, scarring induced by HSV-1 following reactivation from latency is a major cause of CS (30–32). Due to pre-existing immune responses, CS is more likely to occur following recurrence than during primary infection (30, 33). Using a mouse model, we showed that anti-HSV-1 neutralizing antibodies can reduce viral load in the eye and protect mice from eye disease and death. However, this antibody does not completely protect immunized mice from ocular replication and latency reactivation (34, 35). We have also shown that ocular HSV-1 infection suppresses CD80 expression and not CD86. In our previous study, we also investigated the role of CD86 in ocular HSV-1 infection (36). In contrast to the role of CD80 in HSV-1 infectivity, depletion of CD86 in mice significantly increased virus titers in the eye of HSV-1-infected mice. Therefore, mice depleted of CD86 displayed completely opposite results as compared to our present study. Suppression of CD80 expression consequently reduced CD8^+^ T cell responses in the eye and TG of infected mice. Hence, there is an absolute need to control HSV-1 infection at the cellular level.

Our published studies showed that ocular HSV-1 infection suppressed CD80 expression by approximately 80% and consequently CD8^+^ T cell responses in the eye and TG of infected mice (15). The ability of HSV-1 to suppress CD80 expression requires the HSV-1 ICP22 gene and suppression of CD80 by ICP22 may be a mechanism of virus self-survival. As a part of the CD80-HSV-1 interaction, we have demonstrated that (i) HSV-1 ICP22 downregulates the expression of CD80, but not CD86, in the presence or absence of anti-HSV-1 antibodies (15); (ii) recombinant HSV-1 expressing CD80 exacerbates CS in infected BALB/c and C57BL/6 mice (15, 37); (iii) suppression of CD80 by ICP22 is mediated by direct binding of HSV-1 ICP22 to the CD80 promoter (15); (iv) mice ocularly infected with recombinant HSV-1 lacking ICP22 develop enhanced eye disease (18); (v) expression of CD80 by HSV-1 in place of LAT compensates for latency reactivation and anti-apoptotic functions of LAT (37); and (vi) CD80 plays a critical role in increasing inflammatory responses in HSV-1-infected mouse corneas (18, 38). Thus, the most efficient way to decrease latency, recurrent infections, and vision loss is to reduce ocular viral load and accelerate viral clearance without inducing side effects.

Our studies presented above indicated that CD80 may play an important negative role in ocular HSV-1 infection. As proof-of-principle, in this study, we used CD80^−/−^ mice, which lack the CD80 gene, and compared their phenotype with WT control mice following ocular infection with the virulent HSV-1 ocular strain of McKrae virus. Our findings have two main highlights. First, in the absence of CD80, CD80^−/−^ mice have a lower virus replication trend in ocularly infected mice than in control WT mice. Thus, the absence of CD80 in HSV-1-infected mice leads to lower overall virus replication in the eyes. Second, in line with our hypothesis, in the absence of CD80, CD80^−/−^ mice have significantly delayed virus reactivation despite CD80^−/−^ and WT mice having similar levels of latency. In this study, we did not detect significant differences in the levels of eye disease between CD80^−/−^ and WT mice, which is likely due to the absence of spontaneous reactivation in mice. It may thus validate our mice survival data too, which are not significantly different in the WT and CD80^−/−^ mice, but mortality in CD80^−/−^ mice is following a higher trend than in WT mice, which could mean the impairment and function of T cells in CD80^−/−^ mice causing more damage than the functional T cells in WT-infected mice.

It is well established that lower virus replication in the eye may affect reactivation in latently infected mice. During the life of a latently infected individual, the virus can occasionally reactivate, travel back to the eye, and cause recurrent eye disease. Consequently, due to problems associated with recurrent ocular infection, preventing and/or reducing virus replication in the eye and reactivation must be a major goal of controlling ocular HSV-1 infection.

In this study, the absence of CD80 had no effect on gB expression in the corneas or TG of CD80^−/−^ mice or WT-infected control mice other than on day 3 PI in TG. Thus, in the absence of CD80, virus replication is enhanced in the TG of infected mice but does not correlate with latency levels or increased reactivation. CD80 is mainly required for effective T cell function. While it is expressed on antigen-presenting cells, we customized a gene expression panel (see Materials and Methods) to evaluate the absence of CD80 during HSV-1 primary infection. The innate expression of a large number of genes involved in HSV-1 pathogenicity was screened using qRT-PCR. No significant correlation was observed between HSV-1 pathogenicity and expression of CD4, CD8, F4/80, CD11c, NK1.1, Ly6G, IL-1α, IL-1β, IL-2, IL-4, IL-6, IFNα2A, IFNβ, IFNγ, IL-12α, IL-12β, CD80, CD86, CD28, CTLA4, CD1d, perforin, granzymes A and B, PD-L1, CD45, and TNF-α.

Our results show that the absence of CD80 in CD80^−/−^ mice is associated with more CD8^+^ T cells and higher PD-L1 expression in latently infected TG than seen in WT control mice. In the HSV-1 model, CD8^+^ T cells remain latent in TG, but we previously showed that CD8^+^ T cells do not play a major role in latency and reactivation in latently infected mouse TG (39), which is consistent with elevated CD8+ T cells in the TG of latently infected mice in the presence of LAT (22). We have also shown that the ICP22Δ40 mutant does not bind the CD80 promoter, leading to increased CD8^+^ T cells, but not CD4^+^ T cells, in corneas (16). We previously showed that CD80 binds to PD-L1 on DCs (23); however, in the current study, latently infected CD80^−/−^ mice, but not latently infected WT mice, had higher PD-L1 expression in TG and higher CD11c expression in corneas only on day 7 PI (Fig. 9B, *P* < 0.05; Fig. 3A, *P* = 0.0008, respectively). This could be due to the absence of CD80; hence, no binding to PD-L1 on CD11c could justify the increased expression, further confirming our previous study. PD-L1 activity was similar in the absence of CD80 on days 3, 5, and 7 PI in both corneas and TG in WT and CD80^−/−^ mice (Fig. 6, *P* > 0.05).

The results of this study demonstrate that CD80 plays a critical role in virus replication and also adversely affects virus reactivation in infected hosts. Therefore, suppression of CD80 can have a therapeutic effect on reducing virus replication and delaying virus reactivation, which may positively affect the host. The results of this study established a previously undescribed mechanism of viral immune evasion that could be exploited to better manage HSV infection. With the recent failure of large-scale phase III HSV-1 vaccine trials, our approach may lead to a more efficacious vaccine. Consequently, blocking the interaction of HSV-1 with CD80 may temper the immune response, thus reducing HSV-1-induced reactivation and the subsequent ocular disease associated with reactivation.

## MATERIALS AND METHODS

### Mice

Wild-type C57BL/6 and C57-BL/6-CD80^−/−^ mice were purchased from Jackson Laboratory and were bred and maintained in the Cedars-Sinai Medical Center pathogen-free animal facility. Six-week-old male and female WT and CD80^−/−^ mice were used in the study.

### Viruses and cells

The triple plaque-purified WT McKrae HSV-1 strain was used for all experiments in this study. Rabbit skin (RS) cells (used to prepare virus stocks, culture mouse tear films, and determine growth kinetics) were grown in Eagle’s minimal essential media supplemented with 5% fetal bovine serum.

### Ocular infection

Mice were infected with 2 × 10^5^ PFU/eye of McKrae virus as an eye drop in 2 µL of tissue culture media as we described previously (34). Corneal scarification was not performed prior to infection.

### Viral titers from the tears of infected mice

Tear films were collected from 25 mice per group on days 1–7 PI using a Dacron-tipped swab. Each swab was placed in 1 mL of tissue culture medium and squeezed. The amount of virus was determined using a standard plaque assay on RS cells as described (18).

### Monitoring corneal scarring and angiogenesis

The severity of CS lesions in mouse corneas was examined by slit lamp biomicroscopy using a scoring scale of 0, normal cornea; 1, mild haze; 2, moderate opacity; 3, severe corneal opacity but iris visible; 4, opaque and corneal ulcer; and 5, corneal rupture and necrotizing keratitis. The severity of angiogenesis was scored using a system in which a grade of 4 for a given quadrant of the circle represents a centripetal growth of 1.5 mm toward the corneal center. The score of the four eye quadrants was summed to derive the neovessel index (range, 0–16) for each eye at a given time point (40). Each cornea was examined, and the mean ± SEM was calculated for each group.

### *In vitro* explant reactivation assay

Mice were sacrificed on day 28 PI, and individual TG were removed and cultured in tissue culture media as described (37). Media aliquots were removed from each culture daily and plated on RS indicator cells to detect the reactivated virus and to determine the time at which the reactivated virus first appeared in the explanted TG cultures.

### RNA and DNA extraction, cDNA synthesis, TaqMan PCR, and RT-PCR

Corneas and TG from individual mice were isolated on days 3, 5, and 7 PI, while on day 28 PI, TG from latently infected mice were collected for RNA extraction, DNA extraction, or reactivation. Collected tissues were processed as described previously (41). Expression of LAT RNA from latent TG was determined using custom-made LAT primers and probe as follows: forward primer, 5′-GGGTGGGCTCGTGTTACAG-3′; reverse primer, 5′-GGACGGGTAAGTAACAGAGTCTCTA-3′; and probe, 5′-FAM-ACACCAGCCCGTTCTTT-3′ (amplicon length = 81 bp). Levels of gB DNA in latent TG were isolated from homogenized individual TG using the commercially available Dnaeasy Blood &Tissue Kit (Qiagen, Stanford, CA, USA) according to the manufacturer’s instructions. PCR analyses were performed using gB-specific primers: forward primer, 5′-AACGCGACGCACATCAAG-3′; reverse primer, 5′-CTGGTACGCGATCAGAAAGC-3′; and probe, 5′-FAM-CAGCCGCAGTACTACC-3′ (amplicon length = 72 bp). The relative copy number of LAT RNA and gB DNA was calculated using standard curves generated from plasmids pGem5317 and pAc-gB1, respectively, by comparing the normalized threshold cycle (*C_T_*) of each sample to the threshold cycle of the standard curve.

Expressions of primary (days 3, 5, and 7) and latent (day 28) genes were measured using qRT-PCR as follows: (i) CD4 (ABI Mm00442754_m1; amplicon length = 72 bp); (ii) CD8α (ABI Mm01182108_m1; amplicon length = 67 bp); (iii) F4/80 (Mm00802529_m1; amplicon length = 92 bp); (iv) CD11c (Mm00498701_m1; amplicon length = 93 bp); (v) Ly6G (Mm04934123_m1; amplicon length = 113 bp); (vi) NK1.1 (Mm00824341_m1; amplicon length = 92 bp); (vii) IL-2 (Mm00434256_m1; amplicon length = 82 bp); (viii) IL-4 (Mm00445259_m1; amplicon length = 79 bp); (ix) IL-6 (Mm00446190_m1; amplicon length = 78 bp); (x) IFN-γ (Mm00801778_m1; amplicon length = 101 bp); (xi) IFN-α2A (Mm00833961_s1; amplicon length = 158 bp); (xii) IFN-β (Mm00439552_s1; amplicon length = 69 bp); (xiii) CD80 (MM00711660_m1; amplicon length  =  117 bp); (xiv) CD86 (Mm00444540_m1; amplicon length = 91 bp); (xv) CD28 (Mm01253994_m1; amplicon length = 98 bp); (xvi) PD-L1 (Mm03048248_m1; amplicon length = 73 bp); (xvii) CTLA4 (Mm00486849_m1; amplicon length = 71 bp); (xviii) IL-1α (Mm00439620_m1; amplicon length = 68 bp); (xix) IL-1β (Mm00434228_m1; amplicon length = 90 bp); (xx) GzmA (Mm01304452_m1; amplicon length = 59 bp); (xxi) GzmB (Mm00442837_m1; amplicon length = 82 bp); (xxii) perforin (Mm00812512_m1; amplicon length = 95 bp); (xxiii) TNFα (Mm00443258_m1; amplicon length = 81 bp); (xxiv) CD45 (Mm01293577_m1; amplicon length = 73 bp); (xxv) IL-12α (Mm00434169_m1; amplicon length = 58 bp); (xxvi) IL-12β (Mm99999067_m1; amplicon length = 63 bp); (xxvii) CD1d Mm00783541_s1; amplicon length = 142 bp); and (xxviii) PD-1 (programmed death 1; ABI Mm00435532_m1; amplicon length = 65 bp). GAPDH served as an internal control in all experiments as (Mm99999915_g1; amplicon length  =  107 bp) to normalize transcripts. Transcripts in corneas and TG were evaluated on different days in acute and latent stages of infection using commercially available TaqMan Gene Expression Assays (Applied Biosystems, Foster City, CA, USA) with optimized primer and probe concentrations. The 2^−ΔΔ*CT*^ method was used to calculate fold change in gene expression relative to expression in uninfected controls.

### Statistical analysis

For all statistical tests, *P*-values less than or equal to 0.05 were considered statistically significant and are indicated by a single asterisk (*). *P*-values less than or equal to 0.001 are indicated by double asterisks (**). A two-tailed Student’s *t*-test with unequal variances was used to compare the differences between the two experimental groups. A one-way ANOVA test was used to compare the differences among three or more experimental groups. All experiments were repeated at least two times to ensure accuracy.

## Data Availability

All data generated or analyzed during this study are included in the manuscript and supporting files.

## SUPPLEMENTAL MATERIAL

Table S1 jvi.02010-23-s0001.pdf List of non-significant genes in corneas and TG of infected WT and CD80-/- mice on days 3, 5, and 7 PI.

ASM does not own the copyrights to Supplemental Material that may be linked to, or accessed through, an article. The authors have granted ASM a non-exclusive, world-wide license to publish the Supplemental Material files. Please contact the corresponding author directly for reuse.
