# Supplementary material for: Absence of CD80 reduces HSV-1 replication in the eye and delays reactivation but not latency levels
Source: J Virol. 2024 Feb 20;98(3):e02010-23. doi: 10.1128/jvi.02010-23 (PMC10949485; doi:10.1128/jvi.02010-23)
Supplement: Table S1 — List of non-significant genes in corneas and TG of infected WT and CD80-/- mice on days 3, 5, and 7 PI. [file jvi.02010-23-s0001.pdf]

Table S1. List of non-significant genes in corneas and TG of infected WT and $CD80^{-/-}$ mice on days 3, 5, and 7 PI.*

Gene	Cornea									TG								
	**Day 3**			**Day 5**			**Day 7**			**Day 3**			**Day 5**			**Day 7**		
	WT	KO	P	WT	KO	P	WT	KO	P	WT	KO	P	WT	KO	P	WT	KO	P
LyG6	0.06± 0.01	0.03± 0.01	NS	0.04± 0.02	0.02± 0.00	NS	0.05± 0.01	0.17± 0.06	NS	1.76± 0.48	0.31± 0.06	NS	3.46± 0.99	1.54± 0.70	NS	3.10± 0.53	1.17± 0.19	NS
IL-2	0.01± 0.00	0	NS	0.28± 0.06	0.14± 0.03	NS	0.06± 0.01	0.12± 0.03	NS	0.0±1 0.00	0.00± 0.00	NS	0.40± 0.07	0.42± 0.11	NS	0.20± 0.04	0.12± 0.04	NS
IL-4	0.003± 0.002	0	NS	0.02± 0.00	0.03± 0.01	NS	0± 0.00	0.01± 0.00	NS	0.03± 0.00	0.03± 0.01	NS	0.13± 0.02	0.30± 0.09	NS	0.04± 0.01	0.04± 0.01	NS
IFN-α2	0.06± 0.01	0.07± 0.01	NS	0.11± 0.02	0.09± 0.02	NS	0.04± 0.01	0.05± 0.01	NS	0.1± 0.03	0.07± 0.02	NS	0.09± 0.02	0.08± 0.02	NS	0.01± 0.00	0.03± 0.00	NS
IFN-β1	0.06± 0.01	0.06± 0.01	NS	0.52± 0.10	0.19± 0.04	NS	0.02± 0.01	0.05± 0.01	NS	0.30± 0.07	0.10± 0.03	NS	0.92± 0.16	0.98± 0.25	NS	0.05± 0.01	0.05± 0.01	NS
IFN-γ	0.18± 0.04	0.10± 0.05	NS	4.37± 0.87	2.88± 0.59	NS	0.73± 0.17	4.48± 1.28	NS	0.98± 0.24	0.73± 0.31	NS	12.52± 2.04	15.7± 3.70	NS	3.95± 0.75	1.98± 0.36	NS
IL-12α	0.06± 0.01	0.11± 0.02	NS	0.05± 0.01	0.07± 0.02	NS	0.06± 0.01	0.20± 0.04	NS	0.58± 0.12	0.21± 0.04	NS	0.45± 0.11	0.46± 0.16	NS	0.61± 0.11	0.20± 0.03	NS
IL-12β	0.7± 0.12	0.54± 0.09	NS	1.01± 0.19	0.49± 0.09	NS	0.51± 0.09	0.86± 0.15	NS	0.48± 0.09	0.22± 0.09	NS	1.68± 0.29	1.35± 0.28	NS	4.19± 0.68	2.21± 0.41	NS
CD80	0.94± 0.16	0.02± 0.00	NS	1.96± 0.37	0.03± 0.01	NS	0.85± 0.15	0.04± 0.01	NS	1.11± 0.20	0.02± 0.00	NS	3.95± 0.64	0.02± 0.01	NS	4.44± 0.87	0.01± 0.00	NS
CD86	2.08± 0.36	1.15± 0.19	NS	9.24± 1.83	3.14± 0.63	NS	2.13± 0.36	4.36± 0.89	NS	5.40± 1.01	2.49± 0.60	NS	19.06± 3.26	13.2± 2.9	NS	16.2± 3.01	8.95± 1.72	NS
CD28	0.26± 0.04	0.2± 0.04	NS	1.04± 0.21	0.51± 0.11	NS	0.64± 0.14	1.31± 0.26	NS	0.38± 0.07	0.16± 0.03	NS	2.54± 0.46	1.22± 0.25	NS	6.95± 1.40	4.50± 0.95	NS
CTLA4	0.07± 0.01	0.01± 0.00	NS	2.13± 0.43	0.56± 0.13	NS	1.40± 0.32	1.85± 0.49	NS	0.07± 0.01	0.03± 0.01	NS	6.74± 1.19	3.45± 0.77	NS	11.7± 2.57	4.43± 0.93	NS
CD1d	0.20± 0.04	0.24± 0.03	NS	0.32± 0.06	0.19± 0.03	NS	0.28± 0.05	0.49± 0.08	NS	1.61± 0.38	0.24± 0.04	NS	3.07± 0.86	0.94± 0.21	NS	0.94± 0.15	0.65± 0.11	NS

*NS = non-significant.
